# Theoretical Model for a Highly Sensitive Near Infrared Biosensor Based on Bloch Surface Wave with Dirac Semimetal

**DOI:** 10.3390/bios11100390

**Published:** 2021-10-14

**Authors:** Qiwen Zheng, Yamei Liu, Wenguang Lu, Xiaoyu Dai, Haishan Tian, Leyong Jiang

**Affiliations:** 1 School of Physics and Electronics, Hunan Normal University, Changsha 410081, China; qiwen@hunnu.edu.cn (Q.Z.); yamei_cl@163.com (Y.L.); 2 School of Electronic Science and Engineering, National University of Defense Technology, Changsha 410073, China; wenguanglu20@163.com; 3 College of Electrical and Information Engineering, Hunan University, Changsha 410082, China; xiaoyudai@126.com

**Keywords:** biosensor, bloch surface wave, dirac semimetal, photonic crystal

## Abstract

In this work, we present a theoretical model of a near-infrared sensitive refractive index biosensor based on the truncate 1D photonic crystal (1D PC) structure with Dirac semimetal. This highly sensitive near-infrared biosensor originates from the sharp reflectance peak caused by the excitation of Bloch surface wave (BSW) at the interface between the Dirac semimetal and 1D PC. The sensitivity of the biosensor model is sensitive to the Fermi energy of Dirac semimetal, the thickness of the truncate layer and the refractive index of the sensing medium. By optimizing the structural parameters, the maximum refractive index sensitivity of the biosensor model can surpass 17.4 × 10^3^/RIU, which achieves a certain competitiveness compared to conventional surface plasmon resonance (SPR) or BSW sensors. Considering that bulk materials are easier to handle than two-dimensional materials in manufacturing facilities, we judge that 3D Dirac semimetal and its related devices will provide a strong competitor and alternative to graphene-based devices.

## 1. Introduction

Optical biosensors belong to an interdisciplinary field, which includes physics, biology and other scientific fields. The research significance of optical biosensors lies in their ability to transform invisible biological phenomena into measurable or visible physical phenomena. Specifically, they can transform the biological signals which need to be measured into quantifiable optical signals that can be easily measured [1]. As optical biosensors have the characteristics of a small size, high sensitivity, non-contact measurement and fewer required samples, they are widely used in the detection of heavy metal ion [2], dangerous viruses [3], pathogenic microbes [4], biological small molecules [5], drug testing [6], etc. With the development of micro-nano technology and the emergence of advanced photoelectric materials, traditional biosensor technology has been further developed into micro-structures. This kind of optical biosensor is easy to integrate, and has a high sensitivity and anti-interference ability. Therefore, the optical biosensors in micro-structures have been of high interest, such as fiber-coupled structures [7], micro-ring cavity structures [8], photonic crystal structures [9], etc. It is worth mentioning that, because the resonance peak of the surface plasmon resonance (SPR) is very sensitive to small changes in the external environment, high-sensitivity sensors based on SPR technology have been widely studied for a long time. Levy et al. realized a highly sensitive SPR waveguide sensor through the transition of modes. By optimizing the parameters, they achieved a sensitivity of more than 20,000 dB/RIU [10]. Cai et al. modified the grating based on the double-dip methods and obtained a high-order diffraction angle, thus greatly improving the sensitivity of the grating SPR sensor [11]. In the near infrared band, Akter et al. also developed a low-cost, plasmonic refractive index sensor using two-sided open-channels that can operate in both visible and near-infrared regions [12]. In recent years, due to the excellent photoelectric properties of two-dimensional materials, these have been paid more attention. Taking graphene as an example, graphene-gold metasurface SPR biosensors [13], graphene-based fiber optic SPR biosensors [14] and graphene-based D-shaped fiber optic sensors [15] have been reported. In terms of infrared biosensors, Maharana et al. demonstrated an SPR biosensor based on graphene, applied to aluminum and silicon [16]. Sadeghi et al. proposed a graphene-gold ellipse grating SPR biosensor. They claimed that a sensitivity of 1782 nm/RIU and a quality factor of 21,214 were obtained for a refractive index 1.333 [17]. Research into biosensors is still progressing. Although theories and applications regarding the optical biosensors have been almost perfected, achieving optical biosensor schemes with high sensitivity, the adjustable characteristics of their simple structure and dynamics are still a research focus.

Bloch Surface Wave (BSW) is a kind of evanescent wave excited at the interface between truncated PC and homogeneous medium [18]. Unlike the SPR, BSW can be excited by TE or TM polarization [19] and form sharp formants [20,21]. These characteristics mean that BSW has significant advantages in the field of optical sensing. Researchers combined various structures to apply BSW in the field of sensing and found that the sensitivity [22], figure of merit [23], signal-to-noise ratio [24], etc., of biosensors based on the BSW technology are not inferior to classical SPR sensors. Due to the application of mediated electrical materials in photonic crystal, BSW optical biosensors have good chemical and mechanical stability, making them suitable for a variety of harsh environments [25]. Therefore, high-sensitivity BSW biosensors have become a major research focus. For example, Ma et al. obtained the relevance between the wavelength sensitivity and incident angle of a 1DPC structure with an omnidirectional photonic band gap using the Bloch impedance matching method. A sensitivity of 1869 nm/RIU was obtained by optimizing system parameters [26]. Gryga et al. obtained a sensitivity of 1456 nm/RIU by changing the refractive index in the 1–1.005 range, based on the Kretschmann structure, simulated by a coupling prism [27]. In addition, optical biosensors based on two-dimensional materials are currently a research focus in the field of biosensing. Lin et al. realized a high-sensitivity refractive index biosensor by activating BSW based on a prisms–photonic crystal composite structure with graphene, and obtained a sensitivity of 3.5 × 10^4^/RIU through parameter optimization [28]. Zou et al. integrated graphene into a simple Kretschmann structure. By analyzing the performance of the BSW sensor with monolayer graphene, a maximum sensitivity of 3000/RIU was obtained after parameter optimization [29]. In recent years, Bulk Dirac semimetal (BDS) materials [30] have attracted attention. They can be approximately regarded as “3D graphene”, which is similar to graphene and has an adjustable dielectric constant and conductivity [31,32]. Compared with the thin-film structure of graphene, BDS has a larger thickness and a longer propagation length of electromagnetic waves. Researchers believe that the environmental stability of topological Dirac semimetal makes it possible to systematically control and learn [31]. Therefore, it is a good choice for combination with specific mechanisms for sensor designing. Based on the above, in this paper, we propose a BSW biosensor model with a prism-coupled structure, in which BDS is introduced and placed between the dielectric layer and the sensing medium layer. By activating BSW and optimizing the structure parameters, the sensor is highly sensitive to changes in refractive index. In addition, the electrical tunability of BDS’ s conductivity provides a means of achieving sensors with adjustable parameters. We believe this BSW biosensor model, based on BDS, has potential applications in the field of biosensing.

## 2. Theoretical Model and Method

**Figure 1:**
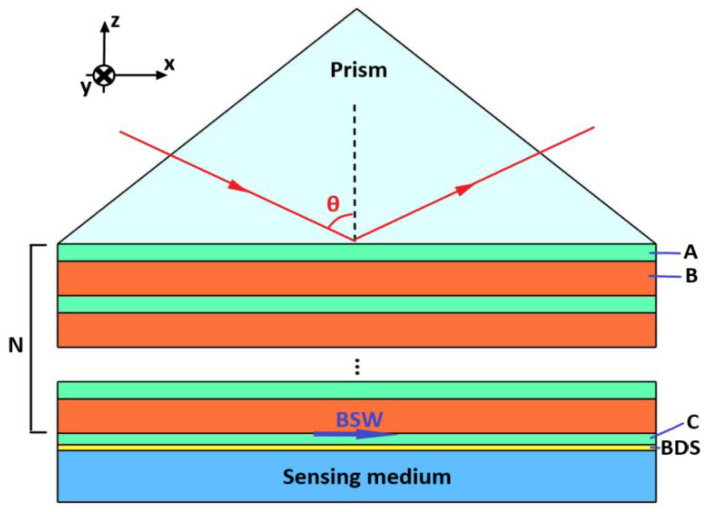
Schematic diagram of BSW sensor based on one-dimensional photonic crystals and BDS.

We consider a prism-coupled BSW sensor structure, as shown in Figure 1. We assumed that the electromagnetic wave was from a prism at an incident angle of *θ*. From top to bottom, the structure was composed of coupling prism, 1D PC, truncate layer, BDS layer and sensing medium layer. Where the refractive index of the prism was expressed as $n_{p}$, 1D PC, composed of alternative A, B, two kinds of different non-magnetic dielectric materials for N cycle structure, its refractive index and thickness, are expressed as $n_{a}$, $d_{a}$, $n_{b}$, $d_{b}$, respectively. In the initial calculation, we set A as TiO_2_ and set B as SiO_2_; the refractive index and thickness were separately taken as $n_{a}=2.3$, $d_{a}=163\;\mathrm{nm}$, $n_{b}=1.434$, $d_{b}=391\;\mathrm{nm}$.

The existence of a truncate layer has a great influence on the sensing performance of the whole structure, so we put the BDS in the truncate layer. In this paper, the truncate layer was composed of dielectric C and BDS, where the corresponding refractive index and thickness of the dielectric material are $n_{c}$, $d_{c}$ respectively; the thickness and refractive index of the BDS material are expressed as $d_{BDS}$ and $n_{BDS}$, respectively, while the refractive index corresponding to the sensing medium layer is expressed as $n_{s}$. We know that the conductivity of the BDS is electrically tunable, so the whole structure’s sensing performance can be made dynamically tunable by adding BDS to the defect layer. The photoelectric characteristics of the BDS were expressed in terms of electrical conductivity; when the low-temperature condition $T\ll E_{F}$ is satisfied, the conductivity of the BDS can be approximated as [33]:

(1) Re(σ(f))=e2ℏgkF24πΩ(f)θ(Ω(f)−2),

(2) Im(σ(f))=e2ℏgkF24π[4Ω(f)−Ω(f)ln(4εc2|Ω2(f)−4|)],

where *g* is the degeneracy factor. In this paper we set *g* = 40 and Fermi velocity as 10^6^ m/s, while $E_{F}$ is Fermi energy level, $k_{F}=E_{F}/\hbar v_{F}$ is Fermi momentum, ℏ is reduced Planck constant, $\tau =\mu E_{F}/v_{F}^{2}$ is electron relaxation time, *μ* is carrier mobility, $\varepsilon_{c}=E_{c}/E_{F}$, and where $\varepsilon_{c}=E_{c}/E_{F}$ is cutoff energy. Based on the above conductivity, the dielectric constant of BDS can be further expressed as

(3) $$\varepsilon_{BDS}=\varepsilon_{b}+i\sigma /\omega \varepsilon_{0},$$

where $\varepsilon_{0}$ is the absolute dielectric constant and $\varepsilon_{b}=1$.

The sensing performance of the scheme requires calculation of the reflectivity of the entire structure. We used the classical and mature transfer matrix method to achieve this. The transfer matrix method (TMM) provides an effective way of solving the reflectivity and transmittance of one-dimensional layered structures. The interaction between medium and light waves can be determined by the characteristic matrix, which can be obtained using Maxwell’s equations to solve the electric and magnetic fields on the adjacent planes $M_{j}$:

(4) $$M_{j}=\left[\begin{array}{cc} \cos\delta_{j} & \frac{i}{\eta_{j}}\sin\delta_{j}\\ i\eta_{j}\sin\delta_{j} & \cos\delta_{j} \end{array}\right],$$

considering the TM and TE polarization, where $\delta_{pj}=\delta_{j}/\cos\theta$, $\delta_{sj}=\delta_{j}\cos\theta$, $\eta_{pj}=K_{j}/\varepsilon_{j}\cos\theta$, $\eta_{sj}=K_{j}\cos\theta /\varepsilon_{j}$. $K_{j}=\sqrt{\varepsilon_{j}-\varepsilon_{0}\sin^{2}\theta }$ is the incident wave vector. For multilayer dielectric structure, the characteristic matrix $M_{total}$ of the whole structure can be obtained by multiplying the M-matrices of each layer. The characteristic equation of the whole structure can be expressed as:

(5) $$\left[\begin{array}{c} B\\ C \end{array}\right]=M_{total}\left[\begin{array}{c} 1\\ \eta_{N+1} \end{array}\right],$$

The above expression can obtain the optical admittance *Y* = *C*/*B* of the structure, and finally obtain the reflectance *R* and transmittance T of the whole structure.

(6) $$\{\begin{array}{c} r=\frac{\eta_{0}-Y}{\eta_{0}+Y}\\ t=\frac{2\eta_{0}}{\eta_{0}+Y} \end{array},$$

(7) $$R={\left|r\right|}^{2},$$

(8) $$T={\left|t\right|}^{2},$$

Sensitivity is the core index when measuring a biosensor’ s performance. In this paper, we mainly discuss the influence on the position of reflection peak when the refractive index of the sensing medium is changing. Therefore, the sensitivity of the sensor can be expressed as:

(9) $$S_{R}=\Delta R/\Delta n_{s},$$

where Δ*n_s_* represents the change in the refractive index in the sensing medium layer, and Δ*R* represents the change in the corresponding reflectivity.

## 3. Results and Discussions

In this section, we discuss the sensing characteristics of the BSW biosensors. Both classical SPR-based biosensors and other biosensors such as Tamm-plasmons-based biosensors need to observe the changes in the reflection peak to sense the characteristics (such as refractive index, etc.) of the sensing medium layer. Our work is similar to those mentioned above. Firstly, we paid attention to the reflectivity curve of the structure shown in Figure 1 as the incident angle changes. In the following calculation, we focused on the biosensing performance of the structure in the near infrared band. The wavelength of the incident light was set as *λ_c_* = 980 nm.The dielectric in the truncate layer was set as TiO_2_, while the corresponding refractive index and thickness were *n_c_* =2.3 and *d_c_* = 120 nm, respectively.

**Figure 2:**
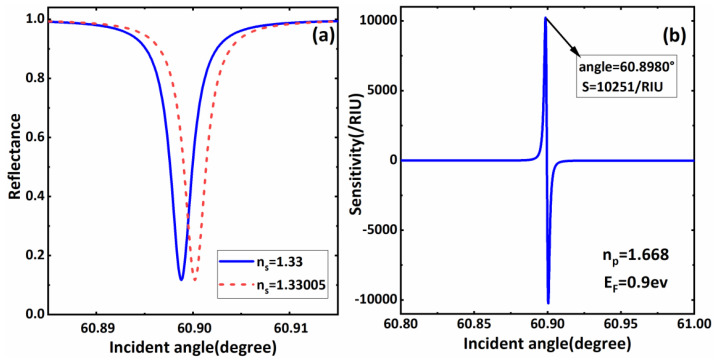
(**a**) The reflection spectrum versus incident angle when *n_s_* = 1.33 and *n_s_* = 1.33005 respectively; (**b**) The sensitivity of sensor varies with incident angle when the refractive index change value of the sensing layer is Δ*n_s_* = 5 × 10^−4^.

We also set the Fermi energy level of the BDS as *E_F_* = 0.9 eV. For the coupling prism, considering that its high dielectric constant requires wave vector compensation to meet the matching condition, which can excite the BSW, the refractive index of the coupling prism is required to be greater than that of the air. Here, we used *n_p_* = 1.668. In addition, we selected the aqueous solution containing biomolecules as the sensing medium layer, and the initial refractive index was *n_s_* = 1.33. Meanwhile, it was assumed that the refractive index of the sensing layer changes was Δ*n_s_* = 0.00005, due to the interaction of biomolecules. We first derived the functional relationship between the reflectivity and incident angle when the refractive index of the sensing medium layer changed, as shown in Figure 2a. According to the figure, when the refractive index of the sensing medium aqueous solution takes *n_s_* = 1.33, an obvious sharp reflection peak appears in the reflectivity curve at 60.898°, which means the excitation of the BSW. This phenomenon has been described in detail in various works and will not be discussed here. On this basis, it was assumed that when the refractive index of the sensing medium layer changes from 1.33 to 1.33005, due to the interaction of biomolecules, the small change in refractive index will lead to a small deviation of the reflection peak in the BSW. Nevertheless, the reflectivity value at the resonance angle was significantly altered by its sharp formant. In this case, the change in value is denoted by Δ*R*. According to the sensitivity calculation formula in the previous section, we can easily obtain the sensitivity of the structure at this time. To more clearly show the sensitivity characteristics near the resonant reflection peak of the BSW, we drew the sensitivity curve near this angle, as shown in Figure 2b. According to Formulas (7) and (8), when the refractive index of the sensing medium layer changes due to the biomolecular interaction, which satisfies Δ*n_s_* = 5 × 10^−4^, the resonance angle has the maximum reflectivity change value Δ*R*, thus corresponding to the maximum sensitivity value. Under the initial structural parameters, set in the previous section, the BSW biosensors proposed in Figure 1 can achieve a sensitivity of greater than 10,000/RIU, which shows a good sensing performance. Therefore, in subsequent discussions, we focus on the sensitivity characteristics at the resonance angle.

**Figure 3:**
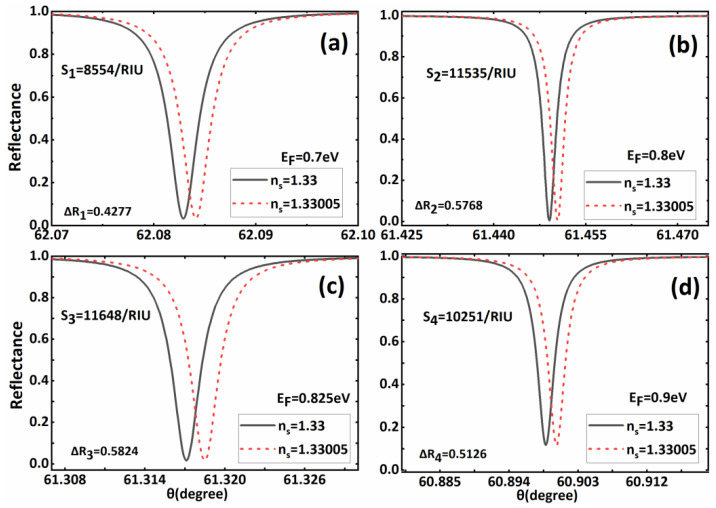
The reflection spectrum versus incident angle when *n_s_* = 1.33 and *n_s_* = 1.33005 respectively with (**a**) *E_F_* = 0.7 eV, (**b**) *E_F_* = 0.8 eV, (**c**) *E_F_* = 0.825 eVand (**d**) *E_F_* = 0.9 eV. The other parameters have the same values as those in Figure 2.

**Figure 4:**
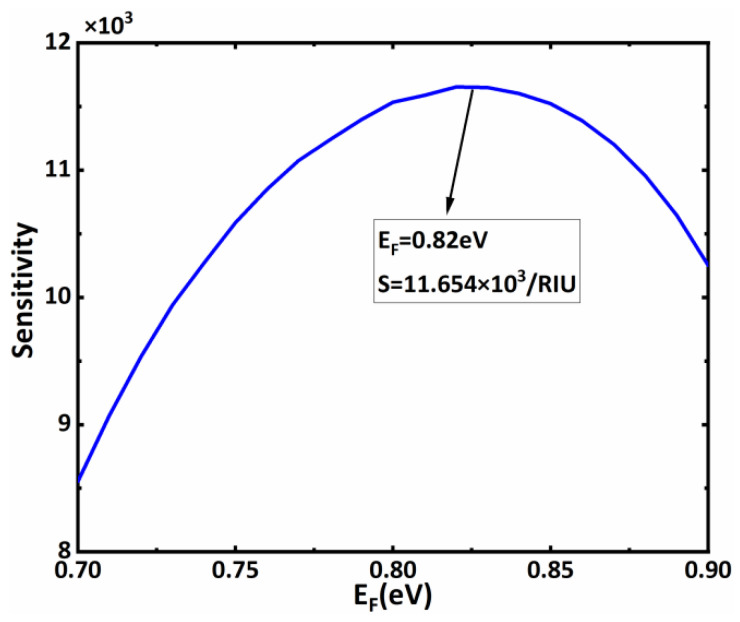
Biosensor’ s sensitivity curve with the Fermi energy level; other parameters are the same as Figure 2.

Based on Figure 2, to further optimize the sensitivity performance of the proposed biosensors, we focus on the influence of the material and structural parameters on the sensitivity of the biosensors in subsequent discussions. From Expressions (1)–(3), it is not difficult to find that the Fermi level of the BDS layer can be dynamically controlled by external voltage, which has a significant influence on the sensing performance of the whole structure. Therefore, we first consider the sensitivity characteristics of the sensor structure under different Fermi energy levels. When the Fermi energy levels are *E_F_* = 0.7 eV, *E_F_* = 0.8 eV, *E_F_* = 0.825 eV, *E_F_* = 0.9 eV, respectively, we drew the curve of the sensor’ s reflectivity as changing with incident angle, as shown in Figure 3. It can be found that, although the Fermi level values in this range do not affect the excitation of the BSW, the corresponding reflection peaks appear, with slight differences between them. First, with the increase in the Fermi level, the resonance angle of the BSW shifts to a lower angle. Secondly, the increase in the Fermi level also results in the change in reflection peak width of the resonance angle of the BSW. These phenomena directly affect the sensitivity value at the resonance angle. Figure 3 also shows that with the increase in the Fermi level, the change in the sensor’ s sensitivity at the resonance angle is not monotonous; therefore, we believe that there is an optimal Fermi level value. To more intuitively find the relationship between the Fermi level and sensitivity, we drew the sensitivity curve at the resonance angle when the Fermi level changed from 0.7 to 0.9, as shown in Figure 4. It is not difficult to observe a maximum value in the sensitivity curve of the sensor with the changes in the Fermi energy level. Specifically, when the Fermi level is in the range of 0.7 eV–0.82 eV, the sensitivity at the resonance angle increases with the rising Fermi level. However, when the Fermi level is in the range of 0.82 eV–0.9 eV, the sensitivity curve decreases. Therefore, when *E_F_* = 0.82 eV, we can obtain the maximum sensitivity value of 11.654 × 10^3^/RIU. In subsequent calculations, we adjusted the Fermi energy level of the BDS to *E_F_* = 0.82 eV, while other parameters remained fixed.

**Figure 5:**
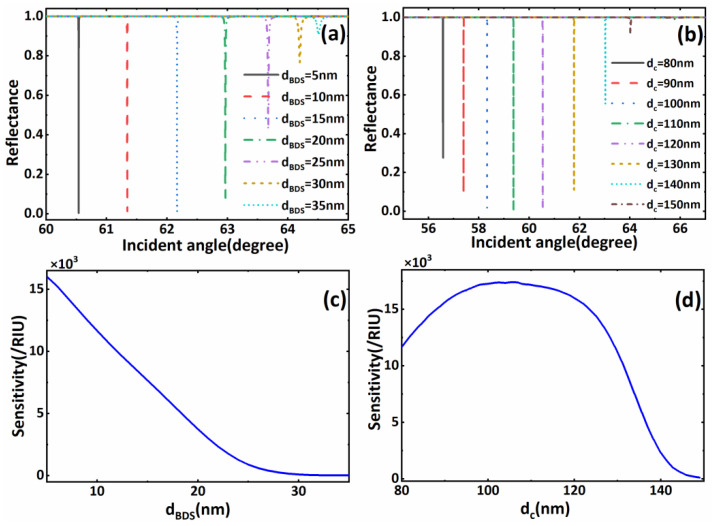
(**a**) The reflection spectrum versus incident angle with different $d_{BDS}$; (**b**) The reflection spectrum versus incident angle with different $d_{c}$; (**c**) The sensitivity curve with $d_{BDS}$ at $d_{c}=120\;\mathrm{nm}$; (**d**) The sensitivity curve with $d_{c}$ at $d_{BDS}=5\;\mathrm{nm}$. The other parameters have the same values as those in Figure 2.

**Figure 6:**
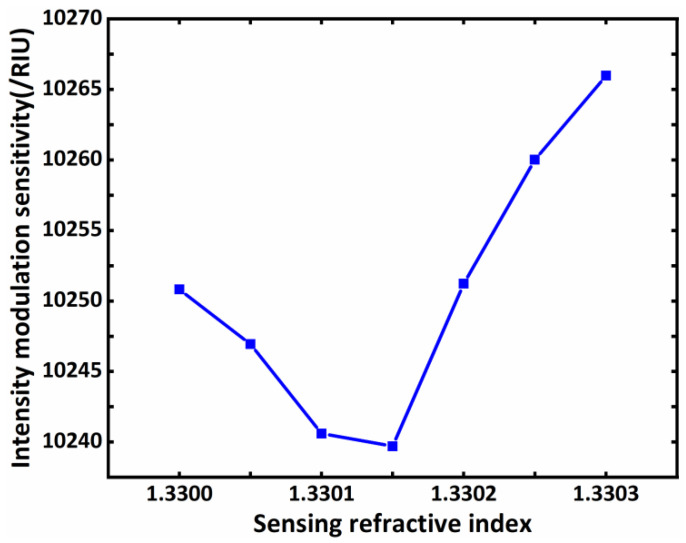
The resonance angle and sensitivity vary with the refractive index of the sensing medium layer. The other parameters have the same values as those in Figure 2.

We know that the location of the reflection peak of the BSW is greatly affected by the defect layer. In this scheme, the sensing medium layer was placed below the defect layer, so it was necessary to study the relationship between the structural parameters of the defect layer and the sensitivity to help optimize the sensing performance. Therefore, we drew the curves of the relationship between the sensitivity of sensor structure and the thickness of the BDS and dielectric material, respectively, as shown in Figure 5a,b. We gradually changed the thickness $d_{BDS}$ of the BDS from 5 nm to 35 nm, and the thickness $d_{c}$ of dielectric material TiO_2_ from 80 nm to 150 nm. According to Formula (4), changing the thickness of each material directly affects their own characteristic matrix, and further regulates the reflectivity curve of the entire structure. It can be seen that with the increasing thickness of the BDS and TiO_2_, the resonance angle starts to shift to the right, and the depth of the formants also changes with the thickness of the BDS and TiO_2_. This change would directly affect the change in sensitivity. Then, we further drew the change in the biosensor’ s sensitivity with the BDS, as shown in Figure 5c. With the increasing $d_{BDS}$, the biosensor’ s sensitivity shows a decreasing trend. When $d_{BDS}=5\;\mathrm{nm}$, we obtained the maximum sensitivity of 16.010 × 10^3^/RIU, and the resonance angle is 60.5398°. In order to further optimize the sensitivity of the sensors, we fixed $d_{BDS}=5\;\mathrm{nm}$ and plotted the change in the biosensor’ s sensitivity with the thickness of TiO_2_, as shown in Figure 5d. With the increasing $d_{c}$, the biosensor’s sensitivity showed a trend first increasing and then decreasing, which provides a reference for us to find and obtain a larger sensitivity value. The above trends are well-reflected in Table 1. This also provides a reference for us for the structural parameters corresponding to the optimized sensitivity. Through the combination of $d_{c}$ and $d_{BDS}$, we found that when $d_{c}=106\;\mathrm{nm}$ and $d_{BDS}=5\;\mathrm{nm}$, the optimized sensitivity value of 17.406 × 10^3^/RIU can be obtained, and the resonance angle is 58.8405° at this time. Finally, we briefly discuss the influence of the refractive index changes in the sensing medium layer on the intensity modulation sensitivity of the biosensors under the initial parameters ($E_{F}=0.9\;\mathrm{eV}$, $d_{c}=120\;\mathrm{nm}$, $d_{BDS}=10\;\mathrm{nm}$). We drew the sensitivity curve of the sensor structure when the refractive index of the sensing medium layer changed slightly, as shown in Figure 6. The sensitivity of the sensor first decreases and then increases with a slight increase in the refractive index of the sensing medium layer. Specifically, when the refractive index of the sensing medium layer increased 1.33–1.33015, the intensity sensitivity of the sensor was slightly reduced with the increasing refractive index of the sensing medium layer. When the refractive index of the sensing medium layer increased by 1.33015–1.3303, the intensity sensitivity was slightly raised by the increased refractive index of the sensing medium layer. Although the slight changes in the refractive index of the sensing medium layer have certain influences on the intensity modulation sensitivity of the biosensor, macroscopically speaking, the influences are limited. As the variation range of the aqueous solution’s refractive index in biosensing is generally very small, and most show a slight increase of 1.33, biological detection in an aqueous solution environment with a refractive index of about 1.33 will generally show a high sensitivity. We also compared our proposed structure with other reported papers and found that our structure has better sensitivity and a simple structure, as shown in Table 2.

## 4. Conclusions

In conclusion, we propose a highly sensitive near-infrared biosensor theoretical model based on BSW. This biosensor scheme excites the BSW using a coupling prism and 1DPC to generate a sharp reflection peak, thus creating the necessary conditions for high sensitivity. By embedding BDS in the defect layer, the sensitivity of the whole structure is enhanced. This also provides dynamically adjustable sensing characteristics. The calculation results show that the sensing performance of the near-infrared biosensor are not only related to the thickness of the defect layer, but are also closely related to the Fermi energy level of the BDS in the defect layer. By optimizing the structure and BDS parameters, we can obtain a maximum sensitivity value of about 17.4 × 10^3^/RIU. At the same time, the theoretical calculation shows that the sensitivity of this biosensor model is at a high level, within the refractive index variation range of the sensing medium layer. Compared to biosensors composed of graphene, this biosensor solution has a simple structure, lower processing requirements, and higher sensitivity. We believe this scheme has potential applications in the field of biosensing based on micro–nano structures.

## Figures and Tables

**Table 1:** Comparison of the sensitivity $S_{R}\;(/\mathrm{RIU})$ with different thickness of *d_BDS_* and $d_{TiO2}$.

	*d_BDS_* = 5 nm	*d_BDS_* = 10 nm	*d_BDS_* = 15 nm	*d_BDS_* = 20 nm	*d_BDS_* = 25 nm	*d_BDS_* = 30 nm	*d_BDS_* = 35 nm
***d_c_* = 80 nm**	11,675	15,342	13,398	10,037	7222	5342	4276
***d_c_* = 90 nm**	15,656	15,362	11,497	8216	6126	5005	4499
***d_c_* = 100 nm**	17,234	14,018	10,001	7450	6066	5167	3684
***d_c_* = 110 nm**	17,159	12,930	9420	7236	5140	2304	443
***d_c_* = 120 nm**	16,010	11,654	7657	3744	899	112	15
***d_c_* = 130 nm**	11,229	5879	1699	240	28	4.83	1.34
***d_c_* = 140 nm**	2324	458	54	7.71	1.82	0.81	0.49
***d_c_* = 150 nm**	72.75	11.43	2.4	0.76	0.52	0.31	0.19

**Table 2:** Comparison between different refractive index sensing methods.

Reference	Mechanism	Structure	Sensitivity	FOM (RIU^−1^)	Frequency Range
[1]	SPR sensor	Photonic crystal fibers (PCFs) structure with open-channels	396/RIU	47	Near Infrared
[2]	SPR biosensor	Aluminum and Silicon-Graphene structure	550/RIU	/	Near Infrared
[3]	Mode coupling sensor	Otto structure	3260/RIU	/	THz
[25]	BSW biosensor	Prism-photonic crystal composite structure with graphene	35,000/RIU	/	Near Infrared
[4]	Mode coupling sensor	Bragg reflector structure(with defect layer)	810 nm/RIU	9679	Near Infrared
[5]	SPR biosensor	Grating- coupled structure	1782 nm/RIU	21,214	Near Infrared
[6]	BSW biosensor	Grating/Bragg mirror structure	128°/RIU	/	Visible
This work	BSW biosensor	Prism-coupled structure(with defect layer)	17,406/RIU	/	Near Infrared

## Data Availability

Not applicable.
